# Testing of Candidate Icons to Identify Acetaminophen-Containing Medicines

**DOI:** 10.3390/pharmacy4010010

**Published:** 2016-01-27

**Authors:** Saul Shiffman, Helene Cotton, Christina Jessurun, Mark A. Sembower, Steve Pype, Jerry Phillips

**Affiliations:** 1 Pinney Associates, 201 N. Craig Street, Suite 320, Pittsburgh, PA 15213, USA; msembower@pinneyassociates.com (M.A.S.); spype@pinneyassociates.com (S.P.); 2 Department of Psychology, University of Pittsburgh, Pittsburgh, PA 15213, USA; 3 Independent market research consultant, Glenview, IL 60026, USA; hcotton@mac.com; 4 McNeil Consumer Healthcare, 7050 Camp Hill Road, Fort Washington, PA 19034, USA; CJessuru@its.jnj.com; 5 Drug Safety Institute, 200 SE 1st St, Miami, FL 33131, USA; jphillips@brandinstitute.com

**Keywords:** acetaminophen, icon, label

## Abstract

Adding icons on labels of acetaminophen-containing medicines could help users identify the active ingredient and avoid concomitant use of multiple medicines containing acetaminophen. We evaluated five icons for communication effectiveness. Adults (*n* = 300) were randomized to view a prescription container label or over-the-counter labels with either one or two icons. Participants saw two icon candidates, and reported their interpretation; experts judged whether these reflected critical confusions that might cause harm. Participants rated how effectively each icon communicated key messages. Icons based on abbreviations of “acetaminophen” (“Ac”, “Ace”, “Acm”) were rated less confusing and more effective in communicating the active ingredient than icons based on “APAP” or an abstract symbol. Icons did not result in critical confusion when seen on a readable medicine label. Icon implementation on prescription labels was more effective at communicating the warning against concomitant use than implementation on over-the-counter (OTC) labels. Adding an icon to a second location on OTC labels did not consistently enhance this communication, but reduced rated effectiveness of acetaminophen ingredient communication among participants with limited health literacy. The abbreviation-based icons seem most suitable for labeling acetaminophen-containing medications to enable users to identify acetaminophen-containing products.

## 1. Introduction

Acetaminophen is an analgesic and antipyretic widely used in both non-prescription (OTC) and prescription (Rx) medicines. An estimated 20% of US adults take acetaminophen in any given week [1] and over 600 US medicines contain acetaminophen [2]. Acetaminophen is safe when used as directed, but overdose can lead to liver injury [2]. Multiple stakeholders, including the Food and Drug Administration, have expressed concern about unintentional acetaminophen overdose [3,4].

Although OTC acetaminophen medicine labels identify acetaminophen as an ingredient and warn against concomitantly using multiple acetaminophen medicines, an important root cause of unintentional overdose is failure to identify acetaminophen as an ingredient and subsequent concomitant use of multiple acetaminophen medicines [5]. A diary study [6] found that concomitant use of multiple acetaminophen medicines was associated with exceeding the maximum daily dose, as was use of multiple medication types (e.g., OTC and Rx) within a day. Individuals with poor knowledge of acetaminophen as an ingredient in their medicines were more likely to exceed the daily limit [5].

Many changes have already been made to medicines labels to facilitate users’ recognition of acetaminophen as an active ingredient and to emphasize the warning against concomitant use [7]. To further facilitate such recognition, it has been proposed that all acetaminophen medicines be marked with an icon that would signal the presence of acetaminophen [8,9]. Graphic communication is commonly used to convey warnings [10,11,12], and has the potential to facilitate rapid recognition, even without extensive reading of the label.

Although education about an icon would be necessary to establish the icon and facilitate its influence on behavior, appropriate design and selection of the icon itself is important to its effectiveness [11]. In other domains, principles and procedures have been developed to guide the development and testing of graphic icons and warnings for consumer products [12]. As the primary purpose of the icon would be to help consumers identify which medications contained acetaminophen, an ideal icon would be readily associated with acetaminophen, lending itself to carrying the intended meaning, and lack pre-existing meanings that could interfere with or undermine the intended communication [12,13]. A particular concern is to avoid icons that might be interpreted in a way that could potentially lead to harm (“critical confusion” [12]), for example if users interpreted the icon to indicate that the medicines bearing it were safe to take together (the opposite of the intended meaning).

This study assesses five candidate icons that were developed through multiple rounds of iterative design and qualitative consumer testing, consistent with guidance for design of safety symbols [12]. This study assesses respondents’ interpretation of the icons for accuracy and critical confusion, as well as their ratings of the icons’ communication effectiveness.

The direct objective of the proposed acetaminophen icon is to facilitate identification of medications in which acetaminophen is the active ingredient, by placing the icon in the active ingredients section of the Drug Facts Label for OTC medicines [14]. The identification of acetaminophen in medications is meant to reduce concomitant use of multiple acetaminophen medications. The icon itself is not intended to or expected to convey this complex message on its own, but it might potentially help draw attention to the concomitant use warning in the label text. With this in mind, it was considered that placing an additional icon in the text warning against concomitant use might improve communication of this warning. This study was a preliminary assessment of consumer response to different candidate icons and icon placements.

## 2. Objectives

The study aimed to assess and compare understanding and misunderstandings of five candidate acetaminophen icons, and to compare a single *versus* dual placement of icons on OTC labels, comparing these to an Rx label.

## 3. Methods

### 3.1. Overview

**Figure 1:**
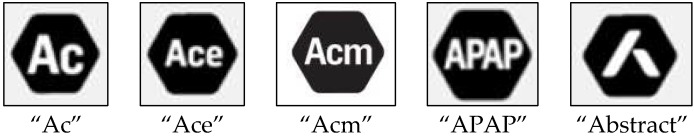
Icons.

The study compared an OTC label with a second icon next to the concomitant use warning to the label with the icon only in the active ingredients section. A third arm tested an icon on a pharmacy Rx container label, on the panel that already carries warnings and icons. Five icon candidates (Figure 1) were tested, across two waves of research, with participants randomized to see either OTC labels with one icon, OTC labels with two icons, or Rx labels with one icon. Some of the methods used were based on those used to test proposed proprietary drug names [15]. Each participant was exposed to two candidate icons, in three contexts: (1) no context; (2) a context (“drug context”) that showed that the icon appeared on a medicine label, but with the label text blurred and not readable; and (3) in the intended context of a readable medicine label (“full label context”). In each context, participants were asked to write in what they thought the icon meant, and what they would do or not do as a result of this interpretation, and their responses were coded for relevant meanings and critical confusion. After reviewing materials that explained the intention of the icon, participants rated the effectiveness of the icon(s) at communicating the key messages of acetaminophen as an ingredient and the warning against concomitant use.

### 3.2. Participants

Participants were 300 adults, participating in two waves of 150 each. The sample was recruited by sending emails to randomly-selected individuals from a panel of 3.8 million people who signed up to participate in online research with Brand Institute. Participants had to be at least 18 years old and English-speaking, and were excluded if they had participated in research in the previous 3 months or if they or an immediate family member worked in healthcare or marketing. By design, the study aimed for 15% of participants to have limited health literacy (14% was achieved), as tested by an online test [16]. To ensure balanced representation of users of the products under study, the samples were stratified to represent by equal proportions those who had and had not used OTC analgesics, antipyretics, or cough/cold products (*i.e.*, those that might contain acetaminophen) in the past 6 months. In Wave 1, a third of the sample was recruited to be current users of anticoagulant medicines. Once the target sample of 150 in each wave had been filled, individuals could no longer enroll; it is not known how many may have wanted to or tried to enroll.

### 3.3. Icon Candidates

Icons were initially developed via an iterative process of graphic design, starting with a pool of candidate approaches, and refining based on consumer response. The candidate icons were developed with feedback from one-on-one qualitative interviews with almost 200 respondents recruited from consumer research panels (about 25% with limited health literacy), via iterative cycles of revision and re-testing. All five icon candidates consisted of a black hexagon (Figure 1), as this developmental qualitative research showed that the hexagon elicited an association with a “stop” sign, causing individuals to interpret the icon as a warning requiring them to pause and pay attention. “Acetaminophen” is an abstract concept not lending itself to concrete or pictorial representation. However, the developmental research suggested that letters contained in the word “acetaminophen” would help people link the icon with this active ingredient. Accordingly, “Ac,” “Ace,” and “Acm” (abbreviation-based icons) were tested, “Ac” and “Ace” in the first wave of the study, and “Acm” in the second wave (to provide an additional abbreviation-based candidate, as “Ac” and “Ace” may potentially have interfering meanings among medical professionals). “APAP” was evaluated in both waves because it is already established among healthcare professionals as an abbreviation for “acetaminophen.” Finally, an abstract symbol (“Abstract”) was evaluated because it was thought least likely to have any pre-existing interfering meaning. Icons were in black and white because many pharmacies cannot print Rx labels in color, and it was considered important for the icon to be presented consistently across OTC and Rx medicine labels.

### 3.4. Procedures

The study was executed in two waves, with procedures identical between waves, but for a few exceptions described below. The study was considered exempt from formal IRB review according to Code of Federal Regulations 45 Part 46.101.b.2, as it was a voluntary and anonymized survey with no sensitive information collected. Participants were recruited without being told that the research concerned medicines, or acetaminophen in particular. Data were collected via the web. Participants indicated, from a list of medicines, what medicines they were taking, so that participants taking anti-coagulants could be unobtrusively identified for Wave 1. Participants completed the Newest Vital Sign [16], a test of health literacy suitable for online administration, and were classified as having limited health literacy if their scores were 3 or less.

**Figure 2:**
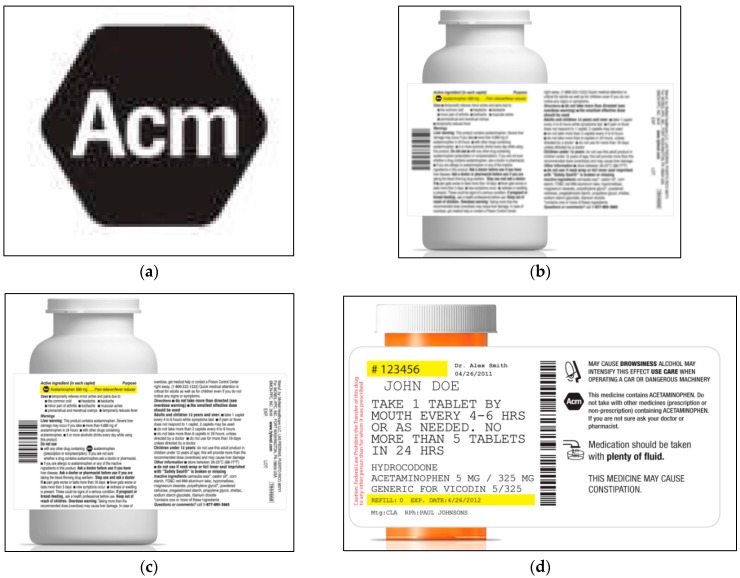
Display of icon in varying contexts and displays. (**a**) No context; (**b**) Drug context (OTC): 1 icon; (**c**) Full label context (OTC): 2 icons; (**d**) Full label context (Rx).

Participants were randomized in equal numbers to one of three icon/label presentations (Figure 2): (1) an OTC label with one icon next to “acetaminophen” in the active ingredients field; (2) an OTC label that added a second icon next to the word “acetaminophen” in the warning against concomitant use (“do not use with any other drug containing acetaminophen”); or (3) an Rx label (for “Vicodin ES”) with the icon and warning in the panel beside the primary panel.

Each participant saw two icon candidates, in random order. In wave 1, all 150 participants saw the “Ac” icon, and were randomized to see one other icon from among “APAP” (*n* = 42), “Ace” (*n* = 55), and Abstract (*n* = 53). In wave 2, all 150 participants saw the same two icons, “Acm” and “APAP.” (Thus, “APAP” was evaluated in both waves, with comparable results.)

Participants were exposed to the icons in three contexts in succession. First, participants saw the icons isolated, with no context (Figure 2a). (We have not included the data on “no context,” as most responses were irrelevant to a medication.) Next, participants saw the icon on a medicine label (“drug context”), but with the label text blurred (Figure 2b); this communicated that the icon appeared on a medicine label, but without readable label text to convey detailed information. Finally, participants saw the icon as it would appear in actual use (Figure 2c,d), surrounded by readable label text (“full label context”). After each presentation, participants wrote in their interpretation of the icon. After viewing the icons in full label context, participants indicated whether the icon was confusing, and were asked to identify the active ingredient from an ingredients list.

After recording their interpretations, participants were shown brief material explaining what acetaminophen is, noting the need to avoid taking too much and to avoid concomitant use of multiple acetaminophen medicines. The text (available as Supplemental material) explained that the icon was intended to signal the presence of acetaminophen and explained the warning against concomitant use. After being exposed to this orientation, participants were asked to rate the icon’s effectiveness in communicating (a) that acetaminophen is an active ingredient; and (b) that multiple acetaminophen medicines should not be used concomitantly.

### 3.5. Measurements

When viewing an icon in each context, participants were asked to write in open-ended text boxes what they thought the icon meant, and what they would do or not do as a result of this interpretation. These responses were coded into thematic categories. Responses indicating the icon meant that acetaminophen was an active ingredient were identified. Responses indicating that the participant would respond by exercising caution (e.g., asking a doctor or reading the label) were also identified and a subset of these indicating avoidance of concomitant use or limiting the acetaminophen dose were also identified. Many responses were idiosyncratic or irrelevant, and did not fit into any common themes; these are tabulated as “other”.

To identify instances of critical confusion, defined as interpretation and behavior that was likely to expose the person to clinical harm [12], verbatim written responses were reviewed, in a blinded manner, by three independent expert judges (See acknowledgements); critical confusion was scored whenever any two judges so indicated.

After responding to the icons in full label context, participants reported whether they found the icon confusing in any way (yes/no). Participants then identified the medicine’s active ingredient(s) from a randomly ordered list of 11 possible ingredients (plus “none of the above” and “don't know”); they were considered correct if they chose “acetaminophen.”

After participants had been exposed to material orienting them to the intended meaning of the icons, they rated each icon on how effective it was at communicating each of two core messages: (1) “that the product contains acetaminophen as an ingredient”; and (2) “that (the user) should not take two acetaminophen products at the same time.” Each was rated on a 1–7 scale, where 1 means “communicates very poorly” and 7 means “communicates very well.” Participants also wrote in the reasons for their ratings.

### 3.6. Analysis

Analyses compared the performance of the five different icons with respect to each of the measures above. Separate analyses, controlling for icon, compared the different icon presentations (1-icon *versus* 2-icon OTC labels, and Rx *vs.* OTC presentations). Also examined were effects of health literacy, including potential moderating or interaction effects. The analyses encompassed data from both waves of the study. The data supported the pooling of data across waves. Participants were drawn from the same pool and recruited in the same way, and the samples did not differ in any demographic factors. Further, responses to the APAP icon, which was assessed in both waves, did not differ between waves, indicating comparability of the data across waves. Because the comparisons involved a mix of within-subjects and between-subjects contrasts, the analysis used Generalized Estimating Equation methods [17,18,19], regression analyses that accommodate this for both dichotomous and quantitative outcomes. Statistical tests used a *p-*value of 0.05.

### 3.7. Results

#### 3.7.1. Recruitment

A total of 10,627 email invitations were sent, of which 40% (4254) bounced back, indicating a currently-invalid email; it is not known how many of the remainder were received or read, nor how many recipients may have tried to enroll after enrollment was closed. Among 1207 individuals who were allowed into the screening phase while the study was open, seven failed to meet inclusion criteria, 631 were excluded because of quotas, and 269 discontinued during screening, yielding the final sample of 300.

#### 3.7.2. Preliminary Analyses

Table 1 shows participants’ demographics. The samples in the two waves did not differ significantly on demographics. Analyses also found no meaningful differences in subject response between those who had and had not used OTC medications in the past 6 months, so detailed analyses by history of use are not reported.

**Table 1:** Demographics.

	Total (%)
	*N* = 300
**Gender**	
Female	50.3%
Male	49.7%
**Race** ^1^	
African American or Black	8.8%
Asian	2.7%
Caucasian or White	77.0%
Hispanic or Latino	8.1%
Other or mixed	3.4%
**Age (years)**	
18–24	6.0%
25–49	49.0%
50–65	37.7%
66–75	6.3%
76+	1.0%
**Employment**	
Full-time	52.3%
Homemaker	13.0%
Part-time	8.7%
Retired	17.3%
Unemployed/seeking employment	8.7%
**Income (annual)** ^2^	
Under $18,000	10.1%
$18,000–$49,999	30.4%
$50,000–$74,999	25.0%
$75,000–$99,999	16.2%
$100,000–$124,999	10.8%
$125,000 or more	7.4%
**Education** ^3^	
Less than High School	2.0%
High School graduate/GED	39.1%
Associate/Bachelor’s degree	46.2%
Post graduate degree	12.7%
**Health Literacy**	
Limited	14.3%
Adequate	85.7%

^1^ Excludes five participants who preferred not to answer; percentages for race based on *N* = 295; ^2^ Excludes four participants who preferred not to answer; percentages for income based on *N* = 296; ^3^ Excludes one participant who preferred not to answer; percentages for education based on *N* = 299*.*

#### 3.7.3. Comparisons among Icons

##### Open-Ended Interpretations

Table 2 summarizes the interpretations made for each icon in drug and full label contexts. In drug context (*i.e.*, on a pill bottle, but without readable label text), a minority of respondents interpreted icons to mean “acetaminophen,” doing so most often for the “Ac” icon. Some additional participants said the icon indicated an active ingredient, without specifying the ingredient. In full label context (with readable label text), most participants interpreted the icons to mean “acetaminophen,” doing so more often for the abbreviation-based icons (about 70%–75% of the time) than for APAP and Abstract (<60%). Persons of limited health literacy were significantly less likely to interpret the icons as acetaminophen (12.8% *vs.* 19.5%; *p* < 0.0001).

**Table 2:** Open-ended interpretation of icons in drug and full label contexts.

	Ac	Ace	Acm	APAP ^1^	Abstract	*p*-Values ^2^		
	*N* = 150	*N* = 55	*N* = 150	*N* = 192	*N* = 53	Icons	Literacy	Interaction
**Open-Ended Interpretation**								
*Drug Context*								
Acetaminophen	30.7% ^a^	18.2% ^b^	14.7% ^b^	14.6% ^b^	9.4% ^b^	0.0125		
By Health Literacy							0.10	0.84
Limited	23.8%	0.0%	9.1%	10.7%	12.5%			
Adequate	31.8%	20.8%	15.6%	15.2%	8.9%			
Other Interpretation:								
*An active ingredient*	5.3%	5.5%	6.0%	6.8%	5.7%			
*Other, drug related ^3^*	23.4%	18.2%	26.7%	20.8%	15.1%			
*Other, not drug*-*related*	5.3%	3.6%	8.0%	4.1%	5.7%			
Don’t know	35.3%	54.5%	44.7%	53.6%	64.2%			
Full Label Context								
Acetaminophen ^6^	75.3% ^a^	76.4% ^a, b^	70.7% ^a, b^	58.9% ^c^	58.5% ^b, c^	0.005		
By Health Literacy							0.0001	0.71
Limited	52.4%	42.9%	36.4%	42.9%	50.0%			
Adequate	79.1%	81.3%	76.6%	61.6%	60.0%			
Other Interpretation:								
*An active ingredient*	6.0%	0.0%	10.0%	6.8%	11.3%			
*Other, drug related ^3^*	10.7%	14.5%	9.3%	11.5%	15.1%			
*Other, not drug-related*	0.7%	0.0%	0.0%	2.1%	0.0%			
Don’t know	7.3%	9.1%	9.3%	20.8%	15.1%			
**Open-Ended Behavioral Response**								
Drug Context								
Would exercise caution ^4^	37.3%	30.9%	40.7%	35.9%	41.5%	0.41		
By Health Literacy							0.92	0.78
Limited	38.1%	28.6%	40.9%	32.1%	37.5%			
Adequate	37.2%	31.3%	40.6%	36.6%	42.2%			
Dose/concomitant use ^5^	0.0%	0.0%	3.3%	3.1%	0.0%	0.40		
By Health Literacy							0.20	0.97
Limited	0.0%	0.0%	0.0%	0.0%	0.0%			
Adequate	0.0%	0.0%	3.9%	3.7%	0.0%			
Full Label Context								
Would exercise caution ^4^	46.7%	52.7%	37.3%	47.9%	50.9%	0.99		
By Health Literacy							0.20	0.58
Limited	28.6%	57.1%	31.8%	39.3%	25.0%			
Adequate	49.6%	52.1%	38.3%	49.4%	55.6%			
Dose/concomitant use ^5^	26.7%	25.4%	20.7%	21.4%	17.0%	0.39		
By Health Literacy							0.56	0.20
Limited	19.1%	14.3%	18.2%	14.3%	12.5%			
Adequate	27.9%	27.1%	21.1%	22.6%	17.8%			

^a, b, c^ Where cells within a row have different superscript letters, this indicates that the two cells differ significantly (*p* < 0.05) from each other. Conversely, cells within a row that share the same superscript letter are not significantly different. For example, in the first row (interpretation as “acetaminophen” in the drug context) the “Ac” icon differs from each of the others. In the seventh data row (interpretation as “acetaminophen” in full label context), “Ace” and “Acm” do not differ significantly compared to either “Ac” or the abstract icon, as they share superscripts (a and b) with those cells. The superscripts are only shown when differences within the row were tested and found significant overall. ^1^ Combines data from Round 1 and Round 2; ^2^ Entries are *p*-values for comparisons across icons (main effect) and health literacy strata (main effect), and their interaction, within that context; ^3^ Includes terms such as “Directions,” “Indication,” “Allergy,” “Other drug,” “Warning.”; ^4^ Includes responses such as “Would ask a doctor,” “Would read the label,” “would avoid taking with another acetaminophen medicine.”; ^5^ Dose/concomitant use is a subset of the percentage of respondents who indicated that they would exercise caution, above. ^6^ For this variable, GEE model-based estimates of the percentages differed substantially from the raw percentages, as follows: AC = 76.1%; ACE = 75.5%; ACM = 70.4%; APA*P* = 57.6%; Abstract = 64.4%.

In drug context, approximately 30%–40% stated they would exercise caution in response to the icons; in full label context, this rose somewhat, with little variation across icons. Whereas almost no respondents interpreted icons as a concomitant use warning in drug context, about 25% did so in label context, with no significant differences across icons.

##### Misinterpretations and Adjudicated Critical Confusion

In drug context, two participants identified “Ace” as meaning “ACE inhibitor”; however, in full label context, no one (including anticoagulant users) interpreted any icon as meaning anti-coagulant or ACE inhibitor. In drug context, two instances of critical confusion were identified: one participant interpreted “Ac” as “aspirin,” and one interpreted “Ace” to mean the medicine would not have side effects or interfere with other medicines. No critical confusion was seen in full label context.

##### Self-Reported Confusion

**Figure 3:**
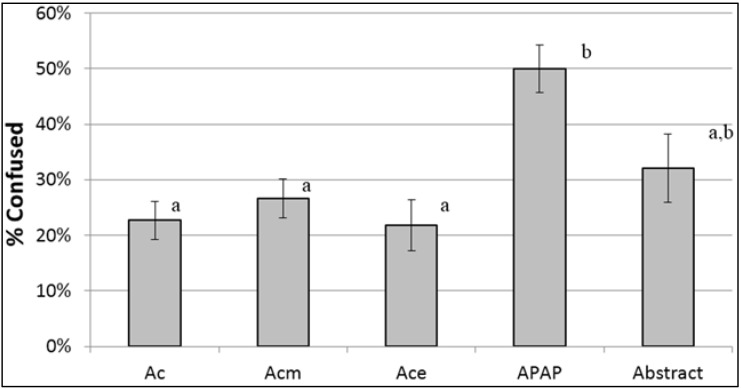
% Confused, by Icon. Letters indicate statistically significant differences: bars that share a common letter do not differ significantly from each other. Thus, the “APAP” icon differs from “Ac,” “Ace,” and “Acm,” which do not differ from each other. The abstract icon did not differ from any of the others.

As shown in Table 3 and Figure 3, the abbreviation-based icons, “Ac,” “Ace,” and “Acm,” were significantly less likely to confuse than APAP, while “Abstract” did not differ from the others. “APAP” was the most often confusing, with half of respondents reporting confusion; respondents indicated this was because the letters did not match those in “acetaminophen.” Limited-literacy respondents were less likely to find icons confusing, across icons.

**Table 3:** Ratings of icons in full label context, overall and by literacy.

	Ac	Ace	Acm	APAP ^1^	Abstract	*p*-Values ^2^		
	*N* = 150	*N* = 55	*N* = 150	*N* = 192	*N* = 53	Icons	Literacy	Interaction
*Confusing (Y/N)*	22.7% ^a^	21.8% ^a^	26.7% ^a^	50.0% ^b^	32.1% ^a, b^	<0.0001		
By Health Literacy							0.05	0.84
Limited	9.5%	14.3%	31.8%	35.7%	0.0%			
Adequate	24.8%	22.9%	25.8%	52.4%	37.8%			
*Correctly Identified Ingredient*	90.0%	92.7%	88.7%	87.0%	84.9%	0.27		
By Health Literacy							<0.0001	0.08
Limited	71.4%	85.7%	63.6%	71.4%	50.0%			
Adequate	93.0%	93.8%	93.0%	89.6%	91.1%			
*Communication Effectiveness (1–7)*								
Acetaminophen as an ingredient	5.83 ^a^ (0.15)	5.76 ^a^ (0.24)	5.76 ^a^ (0.15)	4.28 ^b^ (0.13)	4.72 ^b^ (0.24)	<0.0001		
By Health Literacy							0.35	0.03
Limited	5.67 (0.40)	5.57 (0.67)	5.59 (0.39)	4.79 (0.35)	6.25 (0.63)	0.15		
Adequate	5.86 ^a^ (0.16)	5.79 ^a^ (0.26)	5.79 ^a^ (0.16)	4.19 ^b^ (0.14)	4.44 ^b^ (0.26)	<0.0001		
Concomitant Use	4.67 ^a^ (0.16)	4.47 ^a, b^ (0.25)	5.15 ^a^ (0.16)	4.15 ^b^ (0.14)	4.04 ^b^ (0.25)	<0.0001		
By Health Literacy							0.003	0.88
Limited	5.24 (0.43)	5.29 (0.68)	5.73 (0.42)	4.93 (0.38)	4.88 (0.64)			
Adequate	4.57 (0.17)	4.35 (0.26)	5.05 (0.17)	4.02 (0.15)	3.89 (0.27)			

Note: Means and percentages are raw, unadjusted; standard errors and *p*-values are from GEE models including both icon as the main effect and placement as a covariate; ^a, b^ Where cells within a row have different superscript letters, this indicates that the two cells differ significantly (*p* < 0.05) from each other. Conversely, cells within a row that share the same superscript letter are not significantly different. For example, in the first row (rating the icon as confusing), the “APAP” icon differed significantly from “Ac,” “Ace,” and “Acm”, but not from the abstract icon, with which it shares the superscript b. The abstract icon did not differ significantly from any of the others, with whom it shares the superscripts a or b. The superscripts are only shown when differences within the row were tested and found significant overall; ^1^ Combines data from Round 1 and Round 2; ^2^ Entries are *p*-values. In rows indicating the dependent variable, the *p*-values indicate main-effect differences among icons. When in the row for health literacy, first value is the *p*-value for main-effect differences among participants differing in health literacy.

##### Identification of Acetaminophen as an Ingredient

When viewing the full label, nearly 9 out of 10 participants correctly selected acetaminophen as an active ingredient from a list, with no difference across icons (Table 3). Participants with limited health literacy were less likely to correctly identify acetaminophen as the active ingredient (68.6% *vs.* 91.8%, *p* < 0.0001), across icons.

##### Communication Effectiveness

**Figure 4:**
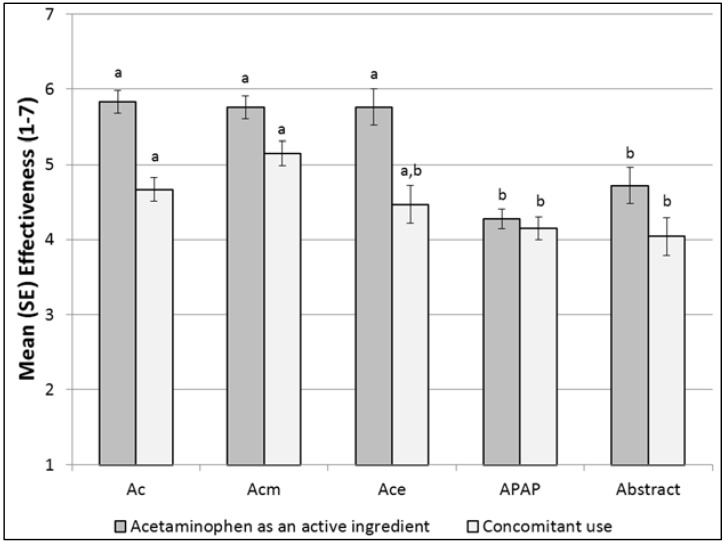
Rated communication effectiveness, by icon. Within the two data series (active ingredient and concomitant use), letters indicate statistically significant differences in communication effectiveness: bars that share a common letter do not differ significantly from each other. For communicating acetaminophen as an active ingredient, “Ac,” “Ace,” and “Acm” differ from “APAP” and the abstract icon. For communicating the prohibition on concomitant use, “Ac” and “Ace” differ from “APAP” and the abstract icon, while “Ace” does not differ from any of the other icons.

Icons differed in rated communication effectiveness (Table 3 and Figure 4). The abbreviation-based icons, “Ac,” “Ace,” and “Acm,” were rated significantly more effective than “APAP” and “Abstract” at communicating acetaminophen as an ingredient. With regard to the concomitant use message, “Acm” and “Ac” were rated higher than “APAP” and “Abstract;” “Ace” was intermediate and did not differ significantly from the others.

Overall, participants with limited health literacy rated communication of concomitant use as more effective than adequate-literacy participants; this was not true for ingredient communication. Health literacy moderated participants’ ratings of the icons’ effectiveness at communicating acetaminophen as an ingredient: Respondents of adequate literacy down-rated “APAP” and (especially) “Abstract” relative to the abbreviation-based icons; low-literacy participants did not.

#### 3.7.4. One *vs.* Two OTC Icons, Rx *vs.* OTC

##### Open-Ended Interpretations

The 1- and 2-icon OTC label presentations were equally likely to elicit caution, but the 2-icon presentation was significantly more likely to elicit specific mention of avoiding concomitant use. Such mentions were also much more likely when viewing the Rx label, compared to the OTC labels (*p* < 0.0001). The icon on the Rx label was less likely than the OTC icon to be interpreted to mean “acetaminophen” in drug context (Table 4), but this effect disappeared when the label was readable.

**Table 4:** Open-ended interpretation of icons in drug and full label contexts, by placement.

	One-Placement OTC	Two-Placement OTC	Rx	OTC Placement: One Icon *vs.* Two Icons (*p*-Values)		Product Class: Rx *vs.* OTC (*p*-Values)	
	*N* = 200	*N* = 200	*N* = 200				
				**Placement**	**Interaction ^2^**	**Class**	**Interaction ^2^**
**Open-Ended Interpretation**							
Drug Context							
Acetaminophen	22.0%	22.0%	11.5%	0.89		0.005	
By Health Literacy ^2^					0.39		0.77
Limited	20.0%	13.9%	0.0%				
Adequate	22.4%	23.8%	12.8%				
Other Interpretations:							
*An active ingredient*	9.0%	6.0%	3.0%				
*Other, drug related*	18.5%	17.5%	30.5%				
*Other, not drug related*	4.5%	7.0%	5.0%				
Don’t know	46.0%	47.5%	50.0%				
Full Context							
Acetaminophen	61.0%	69.0%	72.5%	0.14		0.14	
By Health Literacy ^2^					0.16		0.82
Limited	46.7%	38.9%	50.0%				
Adequate	63.5%	75.6%	75.0%				
Other Interpretations:							
*An active ingredient*	13.5%	5.0%	3.0%				
*Other, drug related*	11.0%	9.0%	14.0%				
*Other*	1.0%	0.0%	1.5%				
Don’t know	13.0%	17.0%	9.0%				
**Open-Ended Behavioral Response**							
Drug Context							
Would exercise caution	36.0%	34.0%	42.5%	0.77		0.14	
By Health Literacy ^2^					0.047		0.52
Limited	53.3%	22.2%	35.0%	0.04			
Adequate	32.9%	36.6%	43.3%	0.59			
Dose/concomitant use ^1^	0.5%	4.0%	1.0%	0.08		0.13	
By Health Literacy ^2^					0.42		0.83
Limited	0.0%	0.0%	0.0%				
Adequate	0.6%	4.9%	1.1%				
Full Context							
Would exercise caution	30.0%	38.0%	69.0%	0.17		<0.0001	
By Health Literacy ^2^					0.94		0.51
Limited	16.7%	25.0%	80.0%				
Adequate	32.4%	40.9%	67.8%				
Dose/concomitant use ^1^	5.5%	14.5%	47.5%	0.03		<0.0001	
By Health Literacy ^2^					0.91		0.17
Limited	0.0%	8.3%	55.0%				
Adequate	6.5%	15.9%	46.7%				

^1^ Dose/concomitant use is a subset of the percentage of respondents who indicated that they would exercise caution, above; ^2^ Main effects of health literacy were previously reported in Table 2.

##### Self-Reported Confusion

Icon placement and product class were not significantly related to reported confusion, and there were no moderating effects of health literacy (Table 5).

**Table 5:** Ratings of icon placements in full label context, overall and by literacy.

	One-Placement OTC	Two-Placement OTC	Rx	OTC Placement: one Icon *vs.* two Icons (*p*-Values)		Placement: Rx *vs.* OTC (*p*-Values)	
	*N* = 200	*N* = 200	*N* = 200				
				**Placement**	**Interaction ^1^**	**Class**	**Interaction ^1^**
**Confusing (Y/N)**	34.5%	32.5%	32.5%	0.52		0.89	
By Health Literacy ^1^					0.10		0.66
Limited	33.3%	13.9%	25.0%				
Adequate	34.7%	36.6%	33.3%				
**Correctly Identified Ingredient**	90.0%	86.0%	84.5%	0.42		0.63	
By Health Literacy ^1^					0.85		0.006
Limited	63.3%	61.1%	80.0%			0.04	
Adequate	94.7%	91.5%	85.0%			0.23	
**Communication Effectiveness (1–7)**							
Acetaminophen as an ingredient	5.37 (0.16)	5.13 (0.16)	5.14 (0.16)	0.45		0.49	
By Health Literacy ^1^					0.025		0.26
Limited	6.33 (0.40)	4.97 (0.36)	4.80 (0.49)	0.02			
Adequate	5.19 (0.17)	5.16 (0.18)	5.18 (0.17)	0.82			
Concomitant Use	4.14 (0.19)	4.37 (0.19)	5.14 (0.19)	0.36		0.0001	
By Health Literacy ^1^					0.49		0.22
Limited	5.30 (0.47)	5.08 (0.43)	5.40 (0.58)				
Adequate	3.94 (0.20)	4.21 (0.21)	5.11 (0.20)				

Note: Means and percentages are raw, unadjusted; standard errors and *p*-values are from GEE models including both placement as the main effect and icon as a covariate and, in cases denoted as such, health literacy status as an additional covariate; ^1^ Main effects of health literacy were previously reported in Table 3.

##### Identification of Ingredient

Placement and product class had no overall effects on identification of acetaminophen as an ingredient (Table 5), but low-health-literacy participants were more likely to identify the ingredient on the Rx label.

##### Communication Effectiveness

The data did not support the hypothesis that adding a second icon placement in the concomitant use warning of the OTC label would enhance communication regarding concomitant use (Table 5). The 2-icon placement was not rated as more effective at communicating regarding concomitant use, nor regarding acetaminophen as an ingredient.

**Figure 5:**
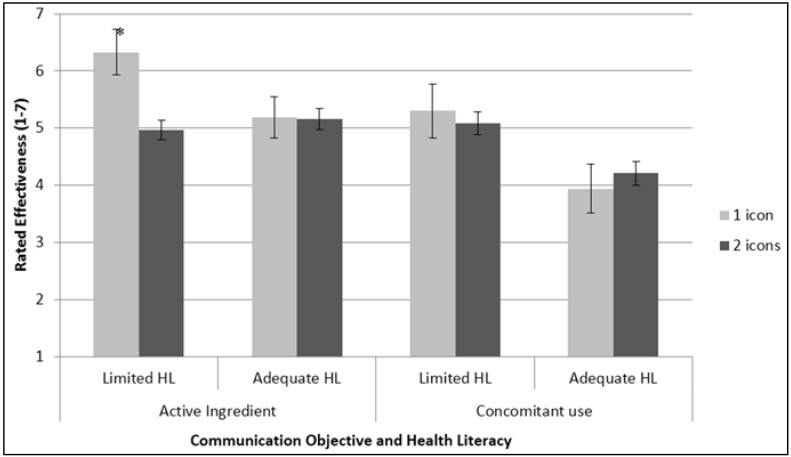
Rated communication effectiveness, one *vs.* two icons, by communications objective and health literacy status. * Statistically significant difference between one and two icons, within the limited literacy stratum. Icon number by literacy status interaction significant for active ingredient communication (*p* < 0.025), but not for concomitant use communication (*p* > 0.25).

Respondents’ ratings of icons’ communication effectiveness regarding concomitant use did not differ by health literacy. However, health literacy did moderate rated communication effectiveness of acetaminophen as an ingredient (*p* < 0.025, as shown in Figure 5). Participants with limited health literacy found the 2-icon presentation significantly less effective than the 1-icon label at communicating acetaminophen as an ingredient (*p* < 0.02); this was not the case for participants with adequate health literacy (*p* > 0.80).

Regardless of health literacy, the Rx label was rated as equally effective to OTC labels at communicating acetaminophen as an ingredient, but more effective than the OTC labels at communicating the warning against concomitant use (*p* = 0.0001; Table 5).

## 4. Discussion

### 4.1. Variation among Icons

A major goal of this research was to identify candidate acetaminophen icons that resonated best with the public. Participants’ responses clearly identified icons that were more and less suitable for communicating about acetaminophen as an ingredient and about the need to avoid concomitant use. The abbreviation-based icons (“Ac,” “Ace,” and “Acm”) generally communicated better than the more abstract ones (“APAP” and “Abstract”).

“APAP”, while historically used by some medical professionals to refer to acetaminophen, but now being phased out of Rx labeling [20], fared poorly. It was rated less effective than all abbreviation-based icons at communicating both key messages: acetaminophen as an ingredient, and avoidance of concomitant use. It was most likely to confuse participants, and was less likely than others to be interpreted as meaning “acetaminophen, “ because participants could not see the connection between the letters “APAP” and the word “acetaminophen”. “APAP” abbreviates the compound’s full chemical name, *N*-acetyl-p-aminophenol. The abstract icon also performed poorly, for similar reasons: participants could not see its connection to acetaminophen. These results were consistent with guidance that suggests avoiding abstract icons [12].

In contrast, the abbreviation-based icons containing letters in the word “acetaminophen” were rated effective in communicating the key messages, were less likely to be deemed confusing, and were more likely to be interpreted as acetaminophen, in part because participants could see how the icon letters related to “acetaminophen”. Although there were minor differences among them, the three abbreviation based icons performed similarly, suggesting that any one of them could usefully serve as an acetaminophen icon.

There had been some concern that the letters “Ac” might be taken to mean “anti-coagulant,” and thereby cause critical confusion. However, this interpretation occurred only once (in a person not on anticoagulants), and the participant interpreted “Ac” correctly as acetaminophen when the label was readable. More broadly, the expert judges reviewing participants’ responses for evidence of critical confusion—confusion that could lead to clinical harm—found none when the icons displayed in the intended context (and none for “Acm,” in any context). Overall, the data suggest that “Ac,” “Ace,” and “Acm” would be suitable icons to communicate about acetaminophen to potential users. The potential for these icons to be misinterpreted by health professionals is being evaluated in a separate study.

### 4.2. Adding a Second Icon to OTC Labels

We also tested the idea that adding a second icon in the OTC warning against concomitant use would improve communication of the need to avoid concomitant use. The data did not consistently bear this out. On the one hand, individuals who saw 2-icon OTC labels were more likely to mention avoidance of concomitant use in their open-ended interpretations. On the other hand, participants did not rate the 2-icon OTC label as more effective at communicating the concomitant use message.

Importantly, adding the second icon appeared to have a down-side: Among participants with limited health literacy, adding the second icon decreased the number of participants who reacted in a cautious way, and reduced the perceived effectiveness of the icon(s) at communicating the core message that acetaminophen was an ingredient, perhaps by diluting the linkage between the icon and the active ingredient. Further research, with careful attention to participants’ health literacy status, may be needed to determine which approach is optimal.

### 4.3. Icons on Rx vs. OTC Labels

The study also compared implementations of the icon on Rx labels *versus* on OTC labels. The Rx implementation was much more likely to be interpreted as a warning against concomitant use, and was also rated more effective at communicating this message. This is not surprising: The Rx label used in this study was visually simpler and less cluttered: it contained 117 words, compared to the 471 words on the OTC label. Importantly, the Rx icon was adjacent to increasingly-standard text noting acetaminophen as an ingredient and warning against concomitant use. On pharmacy container labels, warnings are printed on a designated warnings panel, with ample white space to set off the messages, making the icon and message stand out. In contrast, OTC labels are more densely populated with information required by regulation. The strong performance of the icon on the Rx label is important, as co-use of Rx medicines with OTCs is an important risk-factor for overdose [6], and patients may not expect their Rx medicine to contain acetaminophen.

### 4.4. Effects of Health Literacy

Independent of which icon they saw, participants’ health literacy affected their ability to recognize acetaminophen as an active ingredient. Even when all label text was visible, participants with limited health literacy were less likely to correctly identify acetaminophen as an ingredient from a list of potential ingredients, and also less likely to spontaneously identify the icons as indicating that acetaminophen was the active ingredient. Importantly, as mentioned above, those with limited health literacy rated communication of the most central message—that acetaminophen was an active ingredient—as less effective when a second icon was added to the OTC label. As there is reason to expect that those with limited health literacy may be particularly likely to take too much acetaminophen [21], and may thus be most in need of the guidance an icon may provide, attention to the needs of this population is important, and suggests that a single icon on the active ingredients field of OTC Drug Facts labels may be best.

### 4.5. Limitations

Certain limitations may limit the generalizability and applicability of the results. This study was conducted on-line, likely undersampling persons with very low income, education, or literacy, limiting representativeness. Individuals who volunteered to complete the study may differ from others who did not. However, studies using online panels are often used to collect population data, including on warnings and icons [22,23,24], and are regarded as an appropriate method [25]. Further evaluations of the icon should include the broadest possible range of respondents. Because respondents were online, they could have consulted others or performed online searches to discern the meaning of abbreviations; this would most advantage “APAP”, since it is already in use to mean acetaminophen. Given that the “APAP” icon fared poorly in any case, if respondents did use outside sources, it would only strengthen this result. To examine whether the “Ac” icon might be interpreted as “anti-coagulant”, the sample included some anti-coagulant users. However, the sample did not specifically include users of ACE-inhibitors to assess this sort of confusion with the “Ace” icon. In a previous paper [26], we noted that pharmacists did not mistake the Ace letters in the icon to designate an ACE inhibitor, but concluded, in any case that the possible link to ACE inhibitor drugs made “Ace” a less-than-ideal candidate letter set for an acetaminophen icon.

The study helped identify which icons would be most suitable for communicating about acetaminophen to potential users, but did not test the effectiveness of the icons at facilitating appropriate medication decisions. This needs to be evaluated using other methods. Patient education both about the importance of avoiding concomitant use and about the icon will likely be necessary to make an icon effective in changing behavior.

## 5. Conclusions

Abbreviation-based icons (“Ac,” “Ace,” and “Acm”) were more effective than “APAP” and an abstract icon at communicating that acetaminophen was an ingredient in medicine, and that users should avoid concomitant use with other medicines containing acetaminophen. When seen in a realistic context, most people did not find these icons confusing, and most understood that they designated the active ingredient acetaminophen. Adding a second OTC icon in the warnings section of OTC labels did not consistently improve communication of the warning against concomitant use. Placement of an icon on acetaminophen medicines may help users identify acetaminophen as an ingredient and thus limit concomitant use of acetaminophen medications.

## 6. Practice Implications

The tasks of conveying directions for appropriate use of acetaminophen and motivating appropriate use cannot be accomplished by an icon alone. Users of acetaminophen medications need to be educated about acetaminophen in both Rx and OTC medicines, and about the importance of avoiding concomitant use of multiple acetaminophen medicines. Public education campaigns to inform people about the presence of acetaminophen in multiple medicines, and the need to avoid concomitant use are already under way [27,28], but direct consumer education by pharmacists may also be an important element in educating acetaminophen users. An icon can help individuals recognize acetaminophen medicines, but it needs to be accompanied by efforts by both mass media and healthcare providers to help explain the icon, and convey its importance in guiding users’ behavior. If additional research demonstrates that icons can help the public prevent potential medication errors, there is reason to believe that the addition of an icon to all OTC and Rx acetaminophen medicines in the US could have public health benefit.

## Supplementary Files

Supplementary File 1
