# Supplementary material for: Testing of Candidate Icons to Identify Acetaminophen-Containing Medicines
Source: Pharmacy (Basel). 2016 Jan 27;4(1):10. doi: 10.3390/pharmacy4010010 (PMC5419356; doi:10.3390/pharmacy4010010)

Supplementary Materials: Testing of Candidate Icons to Identify Acetaminophen-Containing Medicines

Saul Shiffman [1,2,*], Helene Cotton [3], Christina Jessurun [4], Mark A. Sembower [1], Steve Pype [1] and Jerry Phillips [5]

This is the display that study participants were shown after they had responded to the icon displays in full label context. It explained the background and intended purpose of the icon, so that participants could evaluate the effectiveness of the icon against this background. Following this display, participants rated the effectiveness of each icon display on how effective it was at communicating each of two core messages: (1) "that the product contains acetaminophen as an ingredient"; and (2) "that (the user) should not take two acetaminophen products at the same time."

Acetaminophen is a medicine that is used to relieve pain and reduce fever. The makers of these medicines are thinking about putting one of the symbols on labels of all the medicines that contain acetaminophen. This is to make it easier for you to recognize when acetaminophen is in the medicine you are about to take, so that you can make sure that you don't take multiple products that contain acetaminophen at the same time.

The example you will see shows the Acm symbol as the example. The makers of these medicines are still deciding which symbol they will actually use on labels of medicines that contain acetaminophen. So, when you answer questions about the other symbols you saw, please imagine that these other symbols appear in the text instead.

Please read the following information:

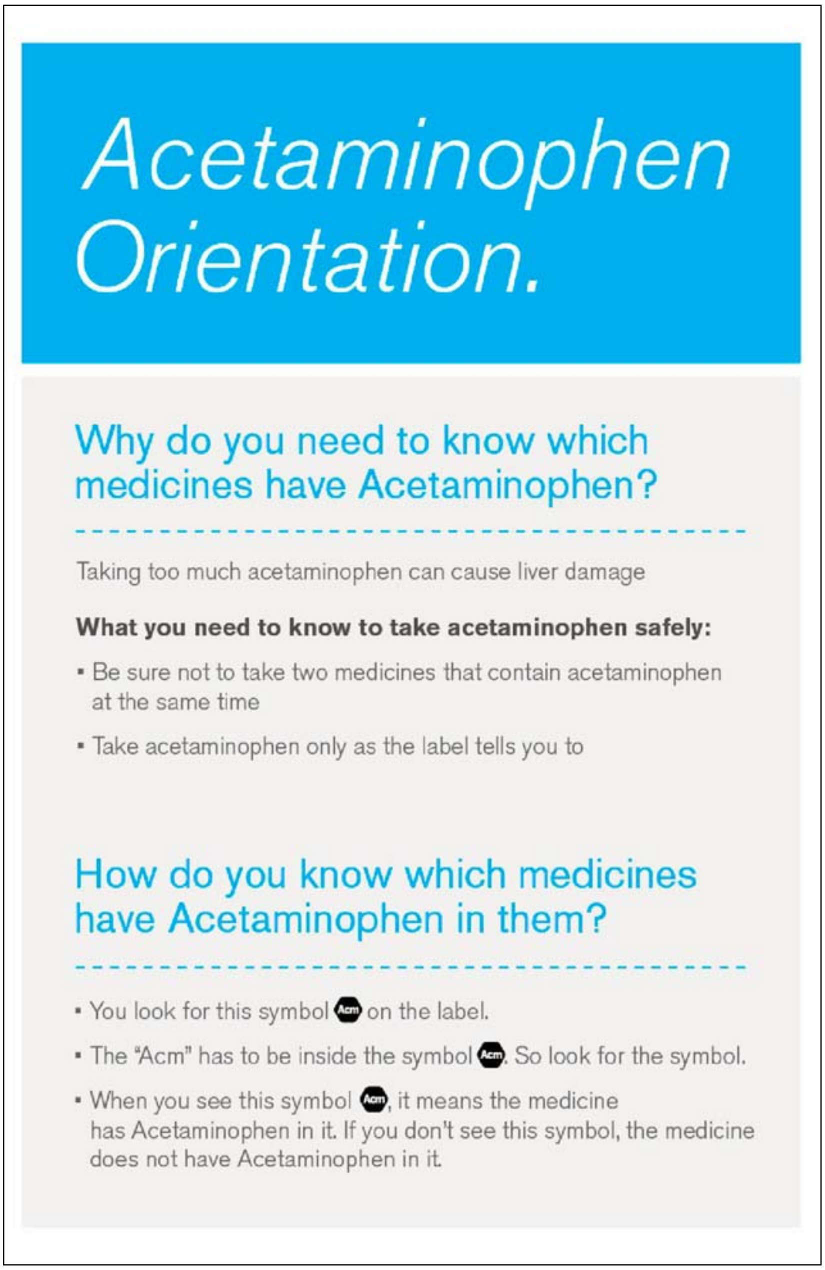

Supplement: Supplementary File 1 [file pharmacy-04-00010-s001.pdf]
